# Immediate Loaded Full-Arch Mandibular Rehabilitations in Younger vs. Elderly Patients: A Comparative Retrospective Study with 7-Year Follow-Up

**DOI:** 10.3390/jcm12134524

**Published:** 2023-07-06

**Authors:** Paolo Capparè, Giulia Tetè, Bianca D’Orto, Matteo Nagni, Enrico Felice Gherlone

**Affiliations:** 1 Dental School, Vita-Salute San Raffaele University, 20132 Milan, Italy; 2 Department of Dentistry, IRCCS San Raffaele Hospital, 00163 Milan, Italy

**Keywords:** elderly patient, edentulism, immediate loading, systemic disease

## Abstract

The aim of this comparative retrospective clinical study was to assess the effect of age on immediate loaded full-arch mandibular rehabilitation in younger vs. elderly patients. Patients with an age between 45 and 60 years (younger group, YG) or with an age more or equal to 75 years (older group, OG), requiring a mandibular full-arch rehabilitation were scheduled for the present study. Implant and prosthetic failure, biological and prosthetic complications, and peri-implant marginal bone level changes were recorded until a 7-year follow-up. Sixty-six patients were included in the study; a total of 264 implants were placed and, in total, 66 “all-on-four” rehabilitations were delivered. In total, 33 patients were scheduled in the YG and 33 patients in the OG. At the 7-year follow-up, an overall implant failure rate of 1.14% was reported. Moreover, at the 7-year radiographic evaluation, peri-implant crestal bone loss averaged 1.12 ± 0.91 mm for the YG and 1.04 ± 1.01 mm for the OG. No statistically significant differences were found between the YG and OG except for the rate of peri-implantitis, which was statistically higher in the YG. The present study reported that immediate fixed mandibular full-arch rehabilitation is a viable procedure in elderly people of equal or more than 75 years of age.

## 1. Introduction

Global demographic trends expect a world population of 9 billion people by the year 2050, with an increase of 50 million annually, probably due to a multitude of factors such as increased life expectancy, reduction in mortality, slow growth, and urbanization [1]. Indeed, it is estimated that in the United States alone, the elderly population (>65 years) will double by the year 2050 [1,2,3,4,5,6,7].

In a clinical study, the authors emphasized that the population will tend to live longer and will, therefore, require endosseous implants to maintain their mandibular overdenture, considering, of course, the economic aspects that could be influential in the choice between removable prosthesis, less expensive prosthesis, and fixed prosthesis [8]. 

In another study, it was suggested that serum factors typical of middle-aged and aged individuals could be responsible, at least in part, for the altered responses observed during wound healing in aging [9]. 

Furthermore, a reduced bone formation response to mechanical loading has been shown with aging and it remains unknown if the interplay between aging and mechanical stimuli during regeneration is similar to adaptation [10,11,12,13]. 

Several studies have been carried out on implant rehabilitation in elderly patients, but they are retrospective for the majority, and the prospective ones involve a small number of patients and mention mainly single implants or conventional rehabilitation [14].

Indeed, a review by Srinivasan et al. reported an implant survival of 97.7% in elderly patients who have performed traditional implant-supported rehabilitation [15]. Other studies showed that there are no significant differences between young and old patients in dental implant outcomes [16,17,18,19,20].

Nevertheless, implant-prosthetic rehabilitation is a highly predictable therapeutical option for partially/completely edentulous jaws, also when implant placement could be limited by anatomical conditions [21]. Indeed, in the atrophic mandible, the anterior region usually maintains an adequate bone volume while in the posterior areas, a severe resorption is present, with a reduced quantity and quality of available bone [22,23,24,25,26]. 

Malò et al. reported, at the long-term follow-ups, that the “all-on-four” is a technique that involves the use of four implants, two axial and two tilted, to avoid anatomical structures at risk without the use of bone grafting procedures [27]. The clinical outcomes of such a procedure, with four implants to rehabilitate an edentulous jaw, were well described in the literature, with favorable 5 to 10-year results [28].

As Cattoni et al. described in 2021, the “all-on-four” digital protocol represents a viable therapeutic choice for implant-supported rehabilitations of edentulous dental arches [29,30,31,32].

Therefore, the aim of this retrospective clinical study was to compare mandibular “all-on-four” rehabilitation outcomes in younger versus older patients at 7-year follow-ups. 

The null hypothesis was that there were statistically significant differences between young and elderly in implant failure, biological and prosthetic complications, and marginal bone loss. 

## 2. Materials and Methods

### 2.1. Patient Selection

This comparative retrospective clinical study was performed at the Department of Dentistry, IRCCS San Raffaele Hospital, Milan, Italy. The ethics committee approval number is 190/INT/2021.

The study was conducted in accordance with the tenets of the Declaration of Helsinki and followed the Strengthening the Reporting of Observational Studies in Epidemiology (STROBE) guidelines for cohort studies (http://www.strobe-statement.org, accessed on 24 April 2021). During the period from January 2014 to December 2021, patients needing full-arch rehabilitation of mandible were evaluated according to the following inclusion criteria:

- All patients had to be edentulous or with only a few hopeless teeth in the mandible and all presented with severe atrophy in posterior regions;
- Sufficient residual bone volume to receive four implants according to the “all-on-four” protocol;
- All patients had to be an age comprised between 45 and 60 years or equal to or more than 75 years;
- All patients had to be independent of help for the activities of daily living;
- All patients had to be in good health.

The exclusion criteria were as follows:

- Patients with contraindications to undergoing implant-prosthetic rehabilitation as severe cognitive impairment (dementia), uncontrolled systemic diseases, taking bisphosphonates [33], or radiation therapy of the head and neck within 1 year;
- Poor oral hygiene;
- Smoking more than 15 cigarettes/day;
- Parafunctional habits (bruxism, clenching);
- Inadequate bone volume;
- Inability to maintain an obligation to implant treatment and maintenance;
- Inability or reluctance to provide informed consent;
- Depression, psychiatric problems, or unrealistic expectations;
- Drug abusers [34];
- Active infection/severe inflammation in the area intended for implant placement [35];
- Participation in other trials if the present protocol could not be properly followed.

Patients with ages between 45 and 60 years were scheduled in the younger group (YG), while patients with ages equal to or more than 75 years were included in the older group (OG).

All diagnoses were made clinically and radiographically. Written informed consent for immediate implant loading was obtained from all patients prior to the implant-prosthetic procedures; professional oral hygiene was provided before surgery. Conventional impressions were taken for study models and temporary prostheses. To assess bone volume (according to Cawood and Howell classification [36]) and bone density (according to Lekholm and Zarb classification [37]) in each patient, the diagnosis was conducted at the first level with orthopantomography and at the second level with CBCT.

### 2.2. Implant-Prosthetic Protocol 

All surgical procedures were done in full safety for the patient’s general health and in consideration of the change of protocols following the COVID-19 pandemic emergency [38,39].

All surgeries were performed by a single experienced surgeon (PC). On the day of surgery, implants were positioned after antibiotic prophylaxis with 2 g of amoxicillin and clavulanic acid (Augmentin, GlaxoSmithKline, Belgium), which was administered 1 h prior to the surgical procedure. The implant surgery was performed under local anesthesia (optocaine 20 mg/mL with adrenaline 1:80,000, Molteni Dental, Florence, Italy).

The implant-prosthetic procedures were performed following Malò’s “all-on-four” protocol [40], already adopted by our Department and described in previous studies [41,42].

The diameter of the final drill was chosen based on bone quality to optimize implant stability. The insertion of the implants followed standard procedures (Winsix, Biosafin, Ancona, Italy), although under-preparation was used in the soft bone to achieve an insertion torque ranging between 30 and 40 N·cm before the final seating of the implant, thereby obtaining high primary stability and immediate function. A manual wrench was also used when incomplete seating of the implant occurred. The implant neck was aimed to be positioned at bone level, and the bicortical anchorage was established whenever possible.

After surgery, mouth rinsing with a chlorhexidine digluconate-containing solution (0.12% or 0.2%), twice a day for 10 days, was prescribed in addition to the recommended standard post-surgical medication: amoxicillin and clavulanic acid (Augmentin, GlaxoSmithKline) 1 g, twice a day for 7 days after surgery and non-steroidal anti-inflammatory drugs (Brufen 600 mg, Abbott Laboratories, Chicago, IL, USA) as needed. All patients were instructed to avoid brushing and any trauma to the surgical site and were recommended to follow a soft diet (avoiding bread and meat) for 2 months. One week after implant placement, sutures were removed [43,44].

After surgery, a low-level laser therapy protocol was performed with a 645 nm diode laser to reduce inflammation of the tissues and to improve the healing phase of the tissues (diode laser, 645 nm, 0, 6 Watt) (EGG Laser, DMT, Lissone, Italy) [45,46,47].

Within 24 h after surgery, provisional full-arch all-acrylic resin prostheses were delivered to all patients based on preliminary impressions. 

In the final prosthesis, the occlusion reproduced the natural dentition with distal cantilevers till the first molar. The pontic areas had an ovate design and the prosthesis provided an intimate contact with the underlying soft tissues but with the cleaning space necessary for the home care oral hygiene. 

In addition to the restoration of esthetics and function, the patient after an implant rehabilitation also improves in their general state of health which is related to an increase in the quality of life [48,49,50,51,52].

### 2.3. Follow-Up

Follow-up visits were performed at 3 and 6 months, then yearly until the 7-year follow-up after implant placement; every 6 months after implant placement, a dental hygienist performed oral hygiene procedures and recorded the clinical parameters, including BI, plaque index, and probing depth around implants [53].

Patients occasionally failed to visit the hygienist but were always recalled for another appointment. 

### 2.4. Outcome Measures

The outcomes considered were as follows: 

1. Implants failure: implant removal dictated by mobility, progressive marginal bone loss due to peri-implantitis, or any mechanical complication rendering the implant not usable (e.g., implant fracture). The stability of each individual implant was assessed manually for 6 months and then yearly from insertion by tightening the abutment screws with the removed prostheses. 

2. Biological and prosthetic complications (number and type) were recorded as single episodes for each implant. Particular attention was used to assess peri-implantitis (defined as a progressive and irreversible disease of hard and soft tissues around dental implants associated with bone loss and signs of infections), presence of pain, presence of pus, paresthesia in the lower jaw, and implant fracture. 

3. Peri-implant marginal bone level changes (MBLCs): Radiographic assessments were made using periapical radiographs obtained immediately after surgery and at each follow-up visit. Bone level measurements were performed on the mesial and distal aspects of each implant using the implant–abutment junction as a reference point; they were made perpendicular to the long axis of the implant with the long-cone parallel technique using an occlusal custom template to measure the MBL. A dedicated dentist measured the changes in crestal bone height over time. The difference in bone level was measured radiographically through custom software (DIGORA 2.5, Soredex, Tuusula, Finland). The software was calibrated for each image using the known implant diameter at the most coronal portion of the neck of the implant. The linear distance between the most coronal point of bone-to-implant contact and the coronal margin of the implant collar was measured to the nearest 0.01 mm at both the mesial and distal sides and then averaged. Marginal bone loss was calculated as the difference in peri-implant bone levels between the first (immediately after fixture placement) and last (during the recall visits) radiographs, and the change in crestal bone height was measured over time. Bone level changes at single implants were averaged at the patient level.

### 2.5. Statistical Analysis

Statistical analysis was carried out using Python 3.8.5 and the following packages: math, sciPy, and pandas. According to the sample distribution, variance, and experimental setting, we used parametric independent samples t-test, Pearson’s chi-square test, or z-test to test for/against differences between groups. Across all analyses, *p*-values < 0.05 were considered significant. Data were analyzed at the aggregate level.

To investigate differences in terms of implant failures and biological and prosthetic complications between groups, Pearson’s chi-square and z-tests were applied at a significance level of *p* < 0.05.

To compare marginal bone loss between groups at 6 months and annually until the 7-year follow-up, Pearson’s chi-square and Student’s *t*-tests were applied at a significance level of *p* < 0.05.

The null hypothesis was that there were no statistically significant differences between the groups compared.

## 3. Results

In total, 87 patients with ages between 45 and 60 years and ages equal to or more than 75 years, needing mandibular full-arch rehabilitations, were screened at the Department of Dentistry, IRCCS San Raffaele Hospital, Milan, Italy. Among them, 66 patients met the eligibility criteria and were included in the study. 

A total of 33 patients were allocated in the YG, while 33 patients were included in OG.

Among them, eight patients (12.90%) were smokers, all from the YG.

According to the “all-on-four” protocol, a total of 264 implants were placed in 66 patients (Table 1) and, in total, 66 “all-on-four” rehabilitations were delivered.

In the YG, 13 patients out of 33 were affected by controlled systemic diseases, while in the OG, 29 out of 33 patients were affected by controlled systemic diseases (Table 2). Moreover, in the YG, two patients were affected by two systemic diseases (hypertension and diabetes in both cases), while in the OG 12 patients were affected by two systemic diseases and three patients by three systemic diseases (Table 2).

In the OG, four drop-outs occurred: one patient died from melanoma at 3.5 years from immediate loading, one patient died from lung cancer at 2 years from immediate loading, one patient died from heart ischemic disease at 1 year from loading and one patient died from a stroke at 6 years and 2 months from loading.

### 3.1. Implant Failure

Implant failure was registered in three patients (3 of 264 fixtures) (Table 3): two implants were lost in the YG and one implant in the OG. Details of the lost implants were reported in Table 3. Moreover, two out of three patients who lost implants were affected by systemic diseases, but no patients were smokers. In each case, implants of the same length and larger diameter were replaced by changing the implant seat. No implant fracture occurred.

So, at the 7-year follow-up, an overall implant failure rate of 1.14% was reported, with a failure rate of 1.52% for the YG and a failure rate of 0.76% for the OG. The differences between the two groups, at a 95% confidence level, appear not to be significant enough to reject the null hypothesis, and the two groups should be considered statistically not different.

### 3.2. Biological and Prosthetic Complications

Peri-implantitis was observed in 11 of 264 implants (4.17%) and 8 of 66 patients (12.12%) (six from YG and two from OG) at the 7-year follow-up. Fractures of provisional prostheses occurred in 4 patients and in 4 of 66 rehabilitations before the 6-month follow-up. In total, two patients were from the YG and two patients were from the OG. No paresthesia and no prosthetic complications in definitive prostheses were registered in any of the patients.

The differences between the two groups, at a 95% confidence level, appear to be significant enough to agree with the null hypothesis, and the two groups should be considered statistically different. Peri-implantitis resulted statistically significantly more frequent in the YG than the OG (*p* = 0.00031). 

### 3.3. Peri-Implant MBLs

MBL outcomes were reported in Table 4. Both axial and tilted implants showed good maintenance of bone levels in both the YG and OG. At the 7-year radiographic evaluation, peri-implant crestal bone loss averaged 1.12 ± 0.91 mm for the YG and 1.04 ± 1.01 mm for the OG. No statistically significant differences in marginal bone loss between Group A and Group B were observed at any of the follow-up evaluations (*p* > 0.05). The differences between the two groups, at a 95% confidence level, appear not to be significant enough to reject the null hypothesis and the two groups should be considered statistically not different. 

### 3.4. Prosthetic Failure

In total, 4 of 66 fixed provisional prostheses were fractured during the observation period, representing a provisional prosthetic fracture rate of 6.01% (Table 5). Among definitive prostheses, no failure was observed, and no fracture of the acrylic resin superstructure occurred.

No statistically significant differences in prosthetic complications (provisional prosthesis fracture, provisional screw loosening (abutment), provisional screw loosening (prosthetic), and detachment of the veneering material (final prosthesis)) between Group A and Group B were observed (*p* > 0.05). The differences between the two groups, at a 95% confidence level, appear not to be significant enough to reject the null hypothesis and the two groups should be considered statistically not different.

## 4. Discussion

This study allows us to compare implant survival between a group of younger and a group of older patients, providing support in the clinical practice of implant rehabilitation according to the “all-on-four” technique. Indeed, the current literature on implants in elderly people focused on overdentures, single implants, and implants in the esthetic zone but no one reported immediate loaded full-arch rehabilitation, despite being a method widely used and endorsed by the scientific literature [15,54].

Malò, in his article on 1070 patients at 13 years of follow-up, says that the “all-on-four” treatment concept is predictable and safe in the long-term outcome [55].

Capparè et al. showed, in their study, that 50 patients received immediately loaded prostheses supported by six implants (a total of 300 implants) with a fixture and prosthetic survival rate of 100% were observed [56].

After all, other authors asserted that definitive cement- and screw-retained ceramic restorations are highly predictable, biocompatible, and esthetically pleasing in full-arch rehabilitation [57]. According to these studies, our results showed that the immediately fixed rehabilitation of the mandible, with two axial and two distal implants, was a suitable procedure, reporting a total implant failure rate of 1.21%.

In contrast with a previous article [58] on systemic patients, in the present study, no smoker patients reported fewer failures than smoker ones; considering its limitations, further clinical trials should compare and analyze this finding.

Another bias is the potential of tissue healing. Indeed, in the review of Srinivasan [15], several biases are emphasized in the elderly population. Aging affects bone healing, therefore osseointegration, and it seems that oral biofilm is altered in aging [59].

In his article, Meyer attributed the possible reason to the fact that older people brush their teeth less and with less care, having a drop in visual and tactile perception and losing oral hygiene as a priority [60].

However, Muller et al. postulated that it is not related to age, but to personal hygiene, so implant rehabilitation should be limited to patients with good oral hygiene and in the absence of bad habits [61].

An overall nonlinear risk pattern of implant failure was observed in the recent study of Jemt which emphasized that middle-aged patients have more risks in implant rehabilitation than young and elderly patients [62]; however, this result may be related to a partial edentulousness of Cawood and Howell I and II classes [22]. 

Moreover, Kower [63] affirmed that dental rehabilitation in partially edentulous elderly patients showed comparable clinical and radiographic results as elderly patients treated in the edentulous jaw. In our study, no significant differences were reported between the younger and older patients, even if peri-implantitis affected younger ones. This is in part according to a recent systematic review [64], which found medium-high evidence that age is not related to peri-implantitis. Therefore, according to our results in full-arch rehabilitations, it could be hypothesized that age can be a secondary variable with respect to the oral hygiene status and bad habits of patients [65]. The null hypothesis was not rejected, except for peri-implantitis, which resulted statistically significantly more frequent in the YG than OG (*p* = 0.00031).

## 5. Conclusions

Within its limitations, the present study reported that immediate fixed mandibular full-arch rehabilitation is a viable procedure in elderly people equal to or more than 75 years of age.

Further studies are needed to evaluate with a longer follow-up period regarding the complications rate, in particular peri-implantitis.

## Figures and Tables

**Table 1:** Implants diameters and lengths.

Mandible n = 264				
		Length 13 mm	Length 15 mm	Length 11 mm
upright n = 132	diameter 3.3 mm	31	0	28
	diameter 3.8 mm	44	0	29
tilted n = 132	diameter 3.3 mm	28	29	0
	diameter 3.8 mm	31	44	0

**Table 2:** Systemic disease for the groups; COPD = chronic obstructive pulmonary disease.

Disease	# Patients YG	# Patients OG
Hypertension	8	18
Diabetes	4	11
Osteoporosis	2	4
Heart failure	2	5
COPD	1	3
Cerebrovascular disease	0	3

**Table 3:** Details of implant failures.

# Patient	Group	Position	Reason of Failure	Time of Failure from Placement	Smoker	Systemic Diseases
1	YG	right mesial	primary infection	1 month	No	No
2	OG	right distal	primary infection	2 months	No	Hypertension and diabetes
3	YG	left mesial	Peri-implantitis	4 years and 3 months	No	Hypertension

**Table 4:** Marginal bone loss for the younger group (YG) and older group (OG).

Bone Loss	YG	OG
6 months (mm)	0.61 ± 0.72	0.62 ± 0.54
1 year (mm)	0.84 ± 0.96	0.80 ± 0.79
2 years (mm)	0.86 ± 0.76	0.82 ± 0.71
3 years (mm)	0.91 ± 0.74	0.86 ± 0.98
4 years (mm)	1.02 ± 1.01	0.90 ± 1.01
5 years (mm)	1.05 ± 0.85	0.93 ± 1.01
6 years (mm)	1.10 ± 0.64	1.03 ±1.04
7 years (mm)	1.12 ± 0.91	1.04 ± 1.00

**Table 5:** Implant failure, prosthetic failure, biological, and mechanical complications.

	# Complications	Rate
Implant failure	4	1.52%
Prosthetic failure	0	0
Fixture fracture	0	0
Peri-implantitis	7	2.65%
Provisional prosthesis fracture	4	6.01%
Episode of pus	0	0
Pain	0	0
Paresthesia	0	0

## Data Availability

Not applicable.
